# Not so ‘rare’—an example of malignant melanoma in India: report from a tertiary cancer centre

**DOI:** 10.3332/ecancer.2021.1335

**Published:** 2021-12-16

**Authors:** Bivas Biswas, Gautam Biswas, Sandip Ganguly, Joydeep Ghosh, Somnath Roy, Manas Kumar Roy, Amrit Pipara, Jagriti Karmakar, Navonil Mukherjee, Santam Chakraborty, Deepak Kumar Mishra, Divya Midha, Deepak Dabkara

**Affiliations:** 1 Department of Medical Oncology, Tata Medical Center, 14 MAR [EW], New Town, Rajarhat, Kolkata 700160, West Bengal, India; 2 Department of Plastic & Reconstructive Surgery, Tata Medical Center, 14 MAR [EW], New Town, Rajarhat, Kolkata 700160, West Bengal, India; 3 Department of GI Surgery, Tata Medical Center, 14 MAR [EW], New Town, Rajarhat, Kolkata 700160, West Bengal, India; 4 Department of Radiotherapy, Tata Medical Center, 14 MAR [EW], New Town, Rajarhat, Kolkata 700160, West Bengal, India; 5 Department of Molecular Oncology, Tata Medical Center, 14 MAR [EW], New Town, Rajarhat, Kolkata 700160, West Bengal, India; 6 Department of Pathology, Tata Medical Center, 14 MAR [EW], New Town, Rajarhat, Kolkata 700160, West Bengal, India

**Keywords:** BRAF, melanoma, India, anorectum

## Abstract

**Purpose:**

Malignant melanoma (MM) is rare in India. Indian data on demography and treatment outcome on advanced MM is very limited in the literature.

**Materials & methods:**

This is a retrospective study of advanced MM treated between January 2013 and December 2020. We evaluated the clinicopathologic features, mutational profiles, survival outcome and prognostic factors in advanced MM patients.

**Results:**

Out of a total 460 patients, 185 (42%) had metastatic disease at presentation and were enrolled in this study with a median age of 63 years (range: 28–93) and male:female ratio of 94:91. The mucosal primary was predominant (*n* = 110, 59%) than cutaneous primary (38%) and anorectum was the most common site (*n* = 84, 45%). Tumour mutational analysis was performed in 65 (35%) patients. *BRAF* mutations were detected in 12 patients and *KIT* mutations in 7 patients. Thirteen patients didn’t have any mutations and 22 patients had mutations other than *KIT & BRAF*. Only 59 (32%) patients took any systemic treatment – immune checkpoint inhibitors (ICIs) in 17, temozolomide in 18 and paclitaxel/carboplatin in 18, tyrosine kinase inhibitors in 6 patients. After a median follow-up of 26 months (95% confidence interval (CI): 11.6–not reached), median progression-free survival (PFS) was 7.1 months (95% CI: 4.4–9.1) and median overall survival was 14.8 months (95% CI: 7.7–18.2 months). The use of ICI emerged as an only significant good prognostic factor (*p* ≤ 0.001) for PFS, on multivariate analysis.

**Conclusion:**

Mucosal origin was more common than cutaneous primary with anorectum being the most common site. *BRAF* mutation was less as compared to published literature. Very few patients received systemic therapy and the use of ICI showed superior PFS.

## Introduction

Malignant melanoma (MM) is an aggressive cutaneous malignancy and ranked 17th in incidence amongst all malignancies globally with a reported annual incidence of 324,635 cases as per Global Cancer Observatory (GLOBOCAN) 2020 data [1]. The incidence of cutaneous melanoma is increasing globally with an estimated 5-year related survival of 93.3% [2]. MM ranked 32nd spot in India as per yearly incidence and recorded 3,916 cases (0.3% of all cases) as per GLOBOCAN 2020 data [3].

Melanocyte exists outside the skin too and can give rise to non-cutaneous MM arising from mucus membrane, uveal tract and leptomeninges [4]. Mucosal MM most often arises from head and neck sinuses and oral cavity, anorectum, vulva and vagina, any other site of the gastrointestinal tract or urogenital tract [5] and constitutes less than 5% of all melanoma cases [6]. The majority of cutaneous MM in western countries presents in the early localised stage (84%) and only 4% presents with advanced stage of disease [7]. The genetic profile of MM varies according to sites and with chronic sun exposure. *BRAF* mutation was highest in patients with MM without chronic sun exposure (56%) whereas* KIT* aberration was mostly found in those of acral, mucosal MM and those with chronic sun exposure (28%–39%) [8].

The survival outcomes of advanced MM have dramatically improved with the discovery of novel targeted therapies like *BRAF* tyrosine kinase inhibitors (TKIs) (like – vemurafenib, dabrafenib) [9, 10] and immune checkpoint inhibitors (ICIs) (nivolumab, pembrolizumab, ipilimumab) [11–13] and established as the standard of care. In comparison, cytotoxic chemotherapy with dacarbazine (DTIC), temozolomide (TMZ) [14] or paclitaxel/carboplatin [15] has shown only a modest and short-lasting response rate.

There is no large-scale publication from India [16–20] about demography, molecular pattern, treatment pattern and/or outcome of MM, and neither any registration trial of novel targeted therapies or immunotherapies has included Indian patients. Here, we have analysed the demography, clinicopathologic feature, tumour mutational profile, treatment pattern including those with novel therapies and outcome of metastatic MM patients treated at our institute.

## Materials & methods

### Patients

This is a retrospective chart review study of all patients ≥ 18 years of age with histologically proven diagnosis of MM treated between January 2013 and December 2020 at our institute. Patients with distant organ metastasis or extensive in-transit metastasis (not amenable for surgical resection) were enrolled for demographic details, clinicopathologic features, treatment details and outcome. A waiver was received from Institutional Review Board because of the retrospective nature of the study.

### Diagnosis

All patients had trucut biopsy or fine-needle aspiration cytology with cell block and demonstration of melanin pigment with appropriate immunohistochemistry (S100, HMB 45 and Melan-A). Spindle cell melanoma (which is mostly amelanotic) was diagnosed with morphology and appropriate immunohistochemistry as mentioned. All patients underwent ^18^Fluorodeoxyglucose positron emission tomography coupled with computed tomography (CT) or contrast-enhanced CT scan of thorax & whole abdomen at baseline. ^99^Technicium bone scan was performed in case of bone related symptoms or elevated serum alkaline phosphatase (in absence of liver metastasis). Central nervous system imaging (either CT scan or magnetic resonance imaging) was performed in symptomatic patients. Baseline lactate dehydrogenase (LDH) was measured for prognostication and as a follow-up tumour marker. Appropriate organ function was measured in those receiving systemic therapy.

### Molecular diagnostics

Every effort was made to perform *KIT* and *BRAF* mutation in all eligible patients. In-house molecular testing started at the end of 2017. *BRAF* mutation was tested by next-generation sequencing (NGS) using Ion Torrent platform with cancer hotspot V2 panel. *KIT* mutation was tested in all patients with mucosal melanoma by NGS. *BRAF* mutation was not performed in patients who don’t have access to anti-*BRAF* TKI.

### Treatment and response

Patients were offered systemic chemotherapy or immunotherapy (preferred option) wherever applicable. Cytotoxic chemotherapy included oral TMZ, inj. DTIC, paclitaxel (conventional or albumin-bound or nanoparticle) with carboplatin. Nivolumab (3 mg/kg every 2 weeks) and pembrolizumab (200 mg every 3 weeks) are approved in India in patients with advanced melanoma. A similar treatment profile was extrapolated for patients with mucosal melanoma. Dabrafenib (150 mg twice daily in empty stomach) & trametinib (2 mg once daily in empty stomach) are the only TKI combination approved in India for advanced melanoma with *BRAF* v600E/K mutation and the same was recommended for patients with *BRAF* v600E/K mutation and a few patients with *BRAF* v600R/D mutation. Appropriate imaging was performed at 2nd or 3rd month of treatment for first response assessment and thereafter as needed or indicated and response measured as complete response (CR), partial response (PR), stable disease (SD) or progressive disease (PD) as per Response Evaluation Criteria in Solid Tumors (RECIST) criteria [21].

### Statistical analysis

Descriptive statistics were used for demographics and clinical characteristics. A Chi-square test was used to detect an association between categorical variables. The student’s *t*-test was applied to compare continuous variables between groups. Survival analysis was performed only in patients who received any form of systemic anti-cancer therapy. Survival was estimated by the Kaplan–Meier method and compared using a log-rank test. Data were censored on 30 April 2021. The Cox proportional hazard model was used in univariate analysis to detect outcome differences between groups. Stepwise multivariate Cox regression analysis was done to identify the predictors of outcome. Factors with significance (*p* ≤ 0.1) in the univariate analysis were entered into multivariate analysis. Progression-free survival (PFS) with the standard error was calculated from the date of starting treatment to the date of disease progression or death due to any cause. Overall survival (OS) with the standard error was calculated from the date of diagnosis to the date of death from any cause. Patients who were lost to follow-up or had treatment abandonment were also included for PFS and OS analysis and the outcome in these patients was confirmed by telephonic contact. Treatment abandonment was included for survival analysis in the present study as it has been proposed that non-compliant and treatment abandonment patients should be included in survival analysis for studies from developing nations to provide a true picture of outcomes from these countries [22]. STATA/SE 11.0 (StataCorp LP, Texas) was used for statistical analysis.

## Results

### Clinicopathologic features

Out of total 460 patients, 185 patients had metastatic disease and were enrolled in this study with a median age of 63 years (range: 28–93; interquartile range: 53–71) and male:female ratio of 94:91. Clinicopathologic characteristics are mentioned in Table 1. The mucosal primary was predominant (*n* = 110, 59%) than cutaneous primary (38%) and anorectum was the most common site (*n* = 84, 45%). Most common sites of metastasis were distant lymph nodes (*n* = 78, 42%) followed by lung (*n* = 67, 36%) and liver (*n* = 66, 36%). More than two sites of organ metastasis were present in 37 (20%) patients. The majority of patients didn’t have baseline LDH and hence were not considered for analysis as a prognostic factor for outcome analysis.

### Tumour mutational profile

**Figure 1.**
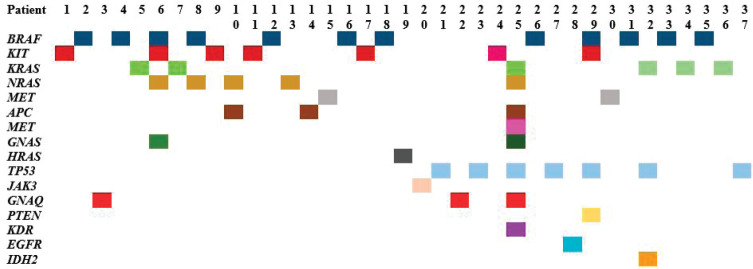
NGS tumour mutational profile of 37 patients with at least one mutation present (*n* = 37).

Tumour mutational analysis was performed in 65 (35%) patients. NGS was performed in 45 patients, *KIT* and *BRAF* sequencing was performed in 5 patients and only *c-KIT* sequencing was done in 15 patients (Table 1). The mutational profile is depicted in Figure 1 and Table 2. Ten patients had *BRAF* mutation (20%, *n* = 50) in v600 codon (v600E in 6, v600R in 3 and v600K in 1 patient) and two had non-v600 codon (N581I and S605I). The *KIT* mutation was present in seven (11%) patients as follows – exon 10 in one, exon 11 in two, exon 13 in two, exon 15 in one and exon 17 in one patient. Thirteen (29%, *n* = 45) patients didn’t have any mutations in NGS testing. Amongst the mucosal melanoma, 34 underwent mutational testing and 4 patients had *BRAF* mutations (3 had v600 codon mutation and 1 non-v600 codon mutation) and another 4 had *KIT* mutations. Twenty-two (49%) patients had mutations other than *KIT & BRAF* which include *NRAS* mutations in five patients (11%).

### Treatment & response

Only 59 (32%) patients took any systemic treatment for their advanced-stage disease (Table 3). Seventeen patients received ICIs as follows – nivolumab in eight patients & pembrolizumab in nine patients. Response to ICI (*n* = 16) was CR in one, PR in seven, SD in five and PD in three patients. Eighteen patients received oral TMZ based therapy and another 18 patients received platinum and taxane combination. Response to chemotherapy was – CR in 1, PR in 3, SD in 15, PD in 15 and status not known in 2 patients. In second line setting, four patients received dabrafenib/trametinib combination therapy (all with *BRAF* v600 mutation) and two patients received nivolumab. Response to dabrafenib/trametinib combination therapy (two in first line setting and four in second line setting) was CR in one, PR in three, SD in one and PD in one patient. Three patients received imatinib as upfront therapy for c-kit mutation and two achieved PRs (exon 11 & exon 13 – one each) and the third patient had clinically PD (exon 13). For brain metastasis, ten patients received whole-brain radiotherapy, two patients underwent surgical resection of the brain lesion and one patient defaulted before any treatment.

### Survival outcome

**Figure 2.**
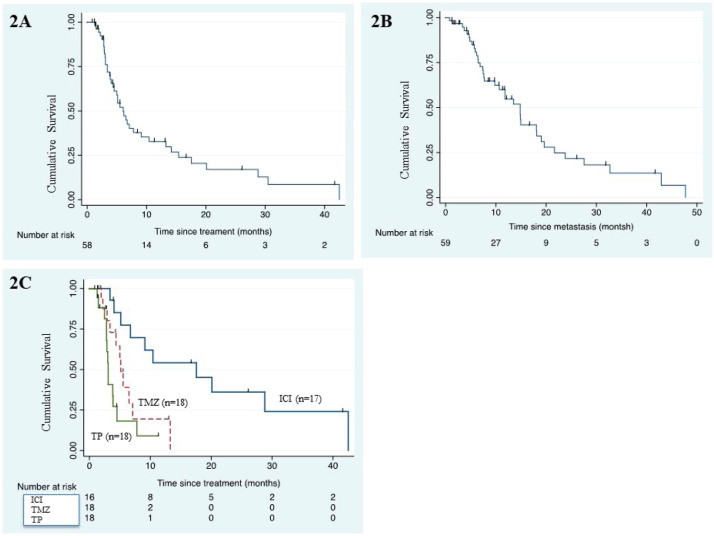
(a): Kaplan–Meier curve for PFS in the whole cohort (*n* = 59). (b): Kaplan–Meier survival curve for OS of the whole cohort (*n* = 59). (c): Kaplan–Meier PFS curve according to type of systemic therapy. (ICI, immune checkpoint inhibitor; TMZ, temozolomide; TP, paclitaxel/carboplatin)

After a median follow-up of 26 months (95% confidence interval (CI): 11.6–not reached), the median PFS was 6.1 months (95% CI: 4.4–9.1) (Figure 2a) and median OS was 14.8 months (95% CI: 7.7–18.2 months) (Figure 2b). The PFS with dabrafenib & trametinib was 6 months & 8 months in first line use and 2, 3, 9, 20 months, respectively, for second line use. The PFS with imatinib was 2, 13.2 & 28.5 months, respectively, for three patients. The median PFS & median OS with first line ICI was 17.6 (95% CI: 5.13–not reached) months and 21.7 months (95% CI: 6.9–42.9), respectively.

### Univariate & multivariate analysis

The univariate analysis of PFS and OS is mentioned in Table 4. Type of systemic therapy (*p* ≤ 0.001) emerged as only significant for PFS (Figure 2c) whereas none of the factors predicted OS.

## Discussion

MM is rare in India [3] and there are few studies on MM regarding its epidemiology, clinicopathologic features, molecular spectrum and treatment outcome due to its rarity. It constitutes 2% of all newly diagnosed solid tumours (460 out of 23,300 cases during the study period) in our hospital as compared to 0.3% cases in India as per GLOBOCAN 2020 data [3]. In our cohort, 185 patients with metastatic disease had predominantly mucosal melanoma, 20% had BRAF mutation, 11% KIT mutation and only one-third took any systemic treatment with a median PFS of 9 months, and one-third of them took an ICI agent. The median age in our cohort was 63 years which was similar to western data [2].

One hundred and ten (59%) patients had mucosal melanoma in our cohort which is a very high incidence as compared to western literature [5] and the majority of the mucosal melanoma was anorectal origin (76%). Both of these observations can be due to the referral bias at our institute being a tertiary care oncology institute. We don’t have any in-house ophthalmic oncology service and this fact may represent very few uveal melanomas in our cohort. It may be a fact that mucosal melanoma incidence is high in India and possibly in the Indian subcontinent as evident by other Indian studies [16, 19, 20]. The high incidence of metastatic disease (185 out of 460) may due to a higher incidence of mucosal melanoma which is very aggressive and presents late, due to delayed referral owing to not much familiarity of the disease entity amongst primary care physicians, etc.

*BRAF* mutation was detected in 20% of the tested population which was much lower as compared to published international data of as high as 56% [8]. This finding may be due to the low sample size as not all patients underwent *BRAF* testing and secondly, a very high incidence of mucosal melanoma in our cohort where the incidence of *BRAF* mutation is very low. *KIT* aberration (mutation) was present in 11% of the tested population and again very low incidence in mucosal melanoma (4 out of 34 tested). Three patients had *BRAF* v600R mutation and two had a long-term response (PFS of 9 months and 20 months, respectively) to dabrafenib/trametinib combination therapy in second line setting (after cytotoxic chemotherapy failure) similar to published literature [23–25].

Only one-third patients (*n* = 59) received systemic therapy owing to many factors – many patients present in very late-stage disease with poor performance status (PS) (*n* = 53, 30% in our cohort), many patients can’t afford immunotherapy (only 16 patients received ICI in first line setting), *BRAF* inhibitor was not available in India during the study period (all received the drug through compassionate access), many didn’t opt for cytotoxic chemotherapy because of dismal outcome (median PFS of 5.1 months and 3.1 months with TMZ and paclitaxel/carboplatin, respectively) and many patients did came for second opinion at our centre after initial treatment elsewhere. Compliance with treatment and access to care is one of the big issues owing to the aforementioned factors in MM patients as we have experienced in our study. Only two [16, 20] of the five Indian studies have reported treatment outcomes (Table 5) in abstract form only and that too mostly with cytotoxic chemotherapy similar to our study results. There is very limited access to clinical trials for MM in India owing to the rarity of the disease in this part of the world.

High serum LDH and ≥3 sites of metastatic disease are well established poor prognostic factors for PFS and OS in advanced/metastatic MM [26, 27]. Mucosal variety has a poor prognosis as compared to cutaneous variety. We didn’t have LDH in the majority of our patients. Metastatic sites or mucosal origin didn’t show any prognostic significance in our series owing to a small number of patients who received systemic therapy. ICI use outperformed other cytotoxic chemotherapy.

There are many limitations in our study. Only one-third of patients took any systemic treatment and many patients lost to follow-up after progression on first line treatment. Hence, prognostication is very difficult with this small sample size. We didn’t have the value of LDH in most of our patients, which is a very important prognostic factor in advanced MM. Our study cohort had referral bias with mucosal primary as the major portion of the population, less head–neck primary but more anorectal primary, very rare cases of uveal melanoma, etc.

## Conclusion

To conclude, melanoma is not that rare in India as reported in GLOBOCAN 2020 database. The majority of our patients had mucosal primary than the cutaneous origin with a very high incidence of metastasis at presentation. Ano-rectal mucosal primary has the maximum incidence probably due to referral bias. Very few patients took systemic treatment and the outcome was determined by the use of ICI over cytotoxic chemotherapy. Access to modern systemic therapy (TKI and ICI) is ‘the’ major concern for the treatment of advanced MM in India owing to the very high cost of the TKI and ICI. Increasing awareness among the primary physician, timely referral to an oncology centre, increasing awareness amongst patients about the disease, multidisciplinary management and improved access to modern systemic therapy may improve current management & outcome of advanced MM in India.

## Ethical clearance

Institute Ethics Committee Protocol Waiver No-EC/WV/TMC/16/21.

## Conflicts of interest

None for each author.

## Funding

Nil.

## Authors’ contributions

Concept and design: BB, SG, SR, DD, SC, AP, DM

Data entry, analysis: BB, SR, JK, NM, JG, DKM, MKR

Manuscript writing, proofreading, final approval: All authors

This study has not been presented in any conference or symposia or elsewhere.

## Figures and Tables

**Table 1.** Baseline clinicopathologic features.

Variables	Number (%)
Age in years (median; range)	63; 28–93
Gender Male Female	94 (51) 91 (49)
Symptom duration in months (median; range)	5; 0.3–60
ECOG PS PS 1 PS 2 PS 3 PS 4	132 (71) 36 (20) 10 (5) 7 (4)
Type of disease Cutaneous Mucosal Uveal + spinal meninges	71 (38) 110 (59) 4 (3)
Site of disease Extremity Head & neck Anorectum Uro-genitals Others	56 (30) 22 (12) 84 (45) 11 (6) 12 (7)
Site of metastasis Distant lymph node Lung Liver Skin & soft tissue Bone Brain^a^	78 (42) 67 (36) 66 (36) 62 (34) 31 (16) 17
Molecular analysis (*n* = 65) NGS *KIT* & *BRAF* sequencing *KIT* sequencing only	45 5 15
Mutation (*n* = 65) *BRAF* *KIT* Others No mutation	12 7 22 13

ECOG, Eastern Cooperative Oncology Group

a Brain imaging performed only in symptomatic cases

**Table 2.** NGS tumour mutational profile of 37 patients with at least one mutation present (*n* = 37).

Mutational profile	Number	%
*BRAF*	9	24%
*BRAF + NRAS*	1	2.5%
*BRAF + KIT + NRAS + GNAS*	1	2.5%
*BRAF + KIT + TP53 + PTEN*	1	2.5%
*KIT*	4	11%1
*KRAS*	4	11%
*KRAS + NRAS + APC + GNAS + TP53 + GNAQ + KRD*	1	2.5%
*KRAS + TP53 + IDH2*	1	2.5%
*NRAS*	2	5%
*NRAS + APC*	1	2.5%
*MET*	2	5%
*APC*	2	5%
*GNAS*	1	2.5%
*HRAS*	1	2.5%
*TP53*	4	11%
*GNAQ*	2	5%
*EGFR*	1	2.5%

**Table 3.** Systemic therapy treatment details.

Agents	Number
	
First line setting (*n* = 59)	
Pembrolizumab	9
Nivolumab	8
TMZ	18
Paclitaxel/carboplatin	14
Nab-paclitaxel/carboplatin	4
Imatinib	3
Dabrafenib/trametinib	2
Crizotinib	1
	
Second line setting (*n* = 17)	
Nivolumab	2
Dabrafenib/trametinib	4
Temozolomide	4
Paclitaxel/carboplatin	5
Nab-paclitaxel/carboplatin	1
Sunitinib	1

**Table 4.** Univariate analysis for PFS & OS (*n* = 59).

		PFS			OS		
Variables	Category (*n*)	HR	CI	*p*	HR	CI	*p*
Age (years)	≤60 (*n* = 31)	1		0.11	1		0.63
	>60 (*n* = 28)	0.59	0.3–1.13		0.85	0.44–1.65	
Gender	Male (*n* = 32)	1		0.87	1		0.86
	Female (*n* = 27)	1.05	0.55–2.01		1.06	0.54–2.06	
Symptoms durations	≤4 months (*n* = 30)	1		0.16	1		0.26
	>4 months (*n* = 29)	0.63	0.33–1.2		0.68	0.34–1.11	
ECOG PS	PS 1 (*n* = 39)	1		0.6	1		0.82
	PS ≥ 2 (*n* = 20)	0.84	0.43–1.62		0.92	0.47–1.82	
Type of disease	Cutaneous (*n* = 20)	1		0.16	1		0.06
	Mucosal/visceral (*n* = 39)	1.64	0.82–3.26		2.02	0.97–4.23	
Metastatic sites	<3 sites (*n* = 148)	1		0.45	1		0.45
	≥3 sites (*n* = 37)	1.33	0.64–2.75		1.36	0.61–3.04	
Treatment types^a^	ICI (*n* = 17)	1		<0.001	1		0.06
	TMZ (*n* = 18)	3.3	1.19–9.2		1.32	0.49–3.55	
	TP (*n* = 18)	6.58	2.36–18.4		2.21	0.95–5.12	

CI, Confidence interval; ECOG, Eastern Cooperative Oncology Group; HR, Hazard ratio; ICI, Immune check point inhibitor; PS, Performance status; TMZ, Temozolomide; TP, Paclitaxel/carboplatin

a Six patients received other systemic therapies

**Table 5.** Published Indian data on advanced MM.

Author (period)	N	Site/type	Molecular features	Chemotherapy	Immunotherapy	Targeted therapy	Median PFS	Median OS
Panda *et al* [18] (2011–2016)	182 Metastatic – 47%	Skin – 93% Mucosa – 7%	NR	NR	NR	NR	NR	NR
Sharma *et al* [19] (1995–2007)	72 Metastatic – 12%	Skin – 35% Mucosa – 25% Viscera – 40%	NR	*N* = 10 Multiple agents	NR	NR	10 months^a^	NR
Mukhopadhyay^b^ *et al* [17] (2016–2019)	93 Metastatic – 86%	Skin – 48% Mucosa – 52%	Not performed	*N* = 39 DTIC – 27 Nab-pacli – 6 Others – 6	*N* = 16 (all nivolumab)	None	3 months	NR
Subhalakshmi (2000–2005)	16 Metastatic – 6%	Skin – 79% Mucosa – 11%	NR	NR	NR	NR	NR	NR
Agarwal^b^ *et al* [16] (2013–2019)	443 Metastatic – 42%	Skin – 41% Mucosa – 57%	*BRAF* – 11%	*N* = 138 (DTIC, taxane, interferon)	*N* = 29	None	5.5 months	11 months
Current study (2013–2020)	460 Metastatic – 185 (40%)	Skin – 38% Mucosal – 59%	BRAF – 20% *KIT* – 11% Others – 49%	TMZ – 18 Taxane/carbo = 18	*N* = 17	*N* = 10^c^	7.1 months	14.8 months

DTIC, Dacarbazine; NR, Not reported; OS, Overall survival; PFS, Progression free survival; TMZ, Temozolomide

a Recurrence free survival in whole population

b Abstract only

c Six patients received dabrafenib/trametinib, three received imatinib and one received crizotinib (in all lines of therapy)
